# Chemical, Rheological and Nutritional Characteristics of Sesame and Olive Oils Blended with Linseed Oil

**DOI:** 10.15171/apb.2018.013

**Published:** 2018-03-18

**Authors:** Fataneh Hashempour-Baltork, Mohammadali Torbati, Sodeif Azadmard-Damirchi, Geoffrey Peter Savage

**Affiliations:** ^1^Department of Food Science and Technology, Faculty of Nutrition, Tabriz University of Medical Sciences, Tabriz, Iran.; ^2^Department of Food Science and Technology, Faculty of Agriculture, University of Tabriz, Tabriz, Iran.; ^3^Food and Drug Safety Research Center, Health Management and Safety Promotion Research Institute, Tabriz University of Medical Sciences, Tabriz, Iran.; ^4^Food Group, Department of Wine, Food and Molecular Biosciences, Lincoln University, Canterbury, New Zealand.

**Keywords:** Nutrition, Rheology, Oil blending, Linseed oil, Olive oil, Sesame oil

## Abstract

***Purpose:*** Nutritional quality and oxidation stability are two main factors in the evaluation of edible oils. Oils in their pure form do not have an ideal fatty acid composition or suitable oxidative stability during processing or storage.

***Methods:*** This study was designed to evaluate the chemical, nutritional and rheological properties of oil mixtures in three ratios of olive: sesame: linseed, 65:30:5; 60:30:10 and 55:30:15. Acidity value, peroxide value, rancimat test, fatty acid profile, nutritional indexes and rheological properties of the oil blends were determined. The nutritional quality was determined by indexes, including the atherogenic and thrombogenic indexs; the ratios of hypocholesterolemic: hypercholesterolemic; poly unsaturated fatty acid: saturated fatty acid and the ω_6_:ω_3_.

***Results:*** The results indicated that blending of other vegetable oils with linseed oil could balance ω_6_:ω_3_. Results showed that formulated oils had a good balance of oxidation stability and nutritional properties as well. Rheological data showed that these oil blends followed Newtonian behavior at 4°C and 25°C.

***Conclusion:*** According to the results, addition of linseed oil to vegetable oils containing high levels of bioactive compounds was a simple and economic practice to obtain a functional oil with good nutritional and stability properties.

## Introduction

Vegetable oils are an important part of our daily diet. Many diseases, such as cardiovascular disease, cholestasis, obesity and other related diseases, are caused by an unbalanced diet particularly in regard to vegetable oils.^1,2^

The nutritional quality and the potential of oils to prevent diseases can be evaluated by five factors: atherogenic index (AI), thrombogenic index (TI), hypocholesterolemic: hypercholesterolemic ratio (HH), polyunsaturated fatty acids: saturated fatty acids (PUFA:SFA) and the ω_6_:ω_3_ ratio, which are calculated from the fatty acids in the oils. AI, TI and H:H are used as predictor factors for cardiovascular disease.^3^

PUFA are essential fatty acids and they can be divided into two groups; omega 3 and omega 6. Based on their structures, each group has important roles in the improvement of the immune system, prevention of cancer and cardiovascular diseases.^4^ Commonly consumed oils, such as sunflower, corn, grape seed and rice bran oils, have high levels of ω_6_ fatty acids and, thus, lead to increases in the ratio of ω_6_ compared to ω_3_ fatty acids. The optimal ratio and balance of these fatty acids has an important effect on their functional properties.^5^ Unfortunately, there is no pure fat or oil that has a balanced amount of essential fatty acids, good oxidative stability and optimum nutritional characteristics. For example, linseed oil is rich in ω_3_ fatty acids, which are easily oxidized, while sesame and olive oils are very stable to oxidation but contain low levels of ω_3_ fatty acids.

Olive oil is one of the most stable oils under heat treatments and deep frying conditions. This characteristic comes from its suitable fatty acid and triacylglycerol composition, phenolic compounds and phytosterols contents.^6,7^

Sesame oil, due to its high level of tocopherols, sesamolin and sesamin lignans, has good oxidation stability and also health promoting effects, such as anti-inflammatory effects and anti-proliferative activity on cancer cells.^8^ Moreover, sesame oil has high amount of ω_6_ fatty acids.^9^Sesame and olive oils, despite all their healthy and nutritious effects, are low in ω_3_ essential fatty acids. Therefore, blending these oils with linseed oil is a good outcome to improve their functional characteristics and increase their applications in the food industry.

Blending vegetable oils with different properties is a simple procedure to create new products with the desired physical, nutritional and oxidative properties and at an affordable price.^10,11^

Rheological measurements can give useful information about the appearance, properties, consistency and sometimes food product quality.^12^ Viscosity means the resistance of fluids moving from one place to another. Thus, fluids with high viscosity need more force to move.^13^ The rheological properties of vegetable oils have new applications in the food industry and play important roles in the determination of their properties particularly when they are used in food formulation and processing.^12,14^

Linseed oil, because of its high content of ω_3_ essential fatty acids and nutritional properties, has received more attention in food formulation. However, linseed oil is very unstable oil and cannot be used alone in food preparations. In this research, linseed oil was blended with olive and sesame oils to obtain a functional oil with high bioactive compounds, optimal essential fatty acids and suitable stability during storage. The present research covers the evaluation of the chemical, nutritional and rheological properties of olive, sesame and linseed oils blends at different ratios and temperatures.

## Materials and Methods

### Materials

The linseed and sesame oils were obtained from seeds using a cold press (screw press, company Iran cold pressing, model 85 mm). Virgin olive oil was purchased from the local market in Tabriz, Iran. The chemical materials used in this study were of analytical grade from Sigma Chemical Co (Sigma Aldrich, St. Louis, MO, USA).

### Methods

#### Blending process

Three formulations of oil blends were olive: sesame: linseed in ratios of: 65:30:5, 60:30:10 and 55:30:15.

#### Acid and peroxide value

Acid value (AV) and peroxide value (PV) of the oil blends were measured by AOAC methods.^15^

#### Oxidative stability

Oxidation stability of the oil samples were determined using the AOCS method Cd12b-92 and a Rancimat instrument model 743 (Metrohm AG., Herisau, Switzerland) was used at 110^o^C with an air flow rate of 20L/h.^16^

#### Fatty acid composition

The oil mixture was converted to fatty acid methyl esters (FAME) by the European Official Methods of Analyzis with a slight modification.^17^ FAME were measured by a gas chromatograph (GC-1000, DANI, Italy) fitted with a flame ionization detector using the method of Azadmard-Damirchi & Dutta.^18^ Identification of the fatty acid profile was undertaken by comparison with chromatograms from reference methyl esters (Sigma Aldrich, St. Louis, MO, USA).

#### Nutritional properties

Nutritional properties were evaluated using different indexes: the atherogenic index (AI), the thrombogenic index (TI) and the hypocholesterolemic: hypercholesterolemic ratio (HH), polyunsaturated fatty acid: saturated fatty acid (PUFA: SFA) and ω_6_:ω_3_ ratio. Calculation of these factors was carried out based on the levels of particular fatty acids using the equations 1 to 3:^19,20^

Equation 1 $$AI=\frac{C_{12:0}+4\times C_{14:0}+C_{16:0}}{\sum MUFA+\sum \bar{\omega }6+\sum \bar{\omega }3}$$

Equation 2 $$TI=\frac{C_{14:0}+4\times C_{16:0}+C_{18:0}}{0.5\times \sum MUFA+0.5\times \sum \bar{\omega }6+3\times \sum \bar{\omega }3}$$

Equation 3 $$HH=\frac{C_{18:1CI39}+C_{18:Z\bar{\omega }6}+C_{20:4\bar{\omega }6}+C_{18:3\bar{\omega }3}+C_{20:5\bar{\omega }3}+C_{22:5\bar{\omega }3}+C_{22:6\bar{\omega }3}}{C_{14:0}+C_{16:0}}$$

#### Rheology

An Anton Paar rheometer (Physica MCR 301, Anton Paar, GmbH, Graz, Austria) was used for the viscosity measurements. The measurements were performed at a shear rate of 1 to 100 s−1 at two temperatures, 4 ± 0.01°C and 25 ± 0.01°C. The Rheoplus Software (version 4.00, Physica MCR series, Anton Parr) was used as the device driver.

Equation 4: The calculation of viscosity and regression coefficient (R^2^) was carried out using Newton’s law equation.

The shear stress to shear rate data was fitted in Newton’s model using linear regression:

Equation 4 = τηγ

Where τ is the shear stress (mPa), γ is the shear rate (s-1) and η is the dynamic viscosity (mPa.s).

*Statistical analysis*

All measurements were carried out in triplicate. The data obtained were analyzed by ANOVA and 16.0 SPSS as statistical software (Chicago, IL, USA) in factorial experiments with an completely randomized design. The results were presented as mean ± standard deviation (SD) of the three measurements. Duncan’s multiple range post hoc test was used to analyze significant differences at the 0.05 level.

## Results and Discussion

### Acidity

Acidity of vegetable oils is a qualitative parameter for determination of triacylglycerol hydrolysis and levels of free fatty acids (FFA). In the formulation of the oil blends, olive oil had the highest acidity among the investigated three oils. During the 90-day storage, all samples showed a significant increase (p < 0.05) in acid value (Table 1). However, it was not high enough to make the oils unusable. According to codex^21^ virgin olive oil can have free fatty acid contents of up to 3.3%. High FFA content of oils makes them prone to oxidation and also reduces the smoking point which makes them less useable in food applications.^22^

Lipase enzymes are one of the most important factors in the hydrolysis of oils and formation of free fatty acids. Furthermore, storage temperature and amount of initial free fatty acids (which can act as catalysts in formation of more free fatty acids) have a significant effect on the acidity value.^23,24^

**Table 1:** Free fatty acid (% oleic acid) and peroxide values (meq O_2_/kg) contents of oil blends during storage up to 90 days (mean ± SD)

**Storage time**	**Parameter**	**Oil blends (olive: sesame: linseed)**		
		**65:30:5**	**60:30:10**	**55:30:15**
Day 1	Free Fatty acid	0.75 ± 0.03	0.71 ± 0.02	0.69 ± 0.01
	Peroxide	5.23 ± 0.25	4.83 ± 0.31	4.63 ± 0.31
Day 30	Free Fatty acid	0.81 ± 0.02	0.78 ± 0.03	0.74 ± 0.02
	Peroxide	6.77 ± 0.26	8.90 ± 0.20	9.40 ± 0.25
Day 60	Free Fatty acid	0.92 ± 0.02	0.89 ± 0.02	0.91 ± 0.02
	Peroxide	9.47 ± 0.15	9.83 ± 0.15	11.10 ± 0.36
Day 90	Free fatty acid	1.08 ± 0.03	1.00 ± 0.02	0.99 ±0.02
	Peroxide	10.97 ± 0.15	13.63 ± 0.15	16.00 ± 0.36
**Analysis of variance**		**df**	**Free fatty acid**	**peroxide**
Storage		3	**	**
Oil blend		2	**	**
Storage× Oil blend		6	*	**
LSD Storage 5%		-	0.0213	0.25
LSD Oil blend 5%		-	0.0185	0.21
LSD Storage × Oil blend 5%		-	0.0369	0.43

** P < 0.01; * P < 0.05 LSD: least significant difference.

### Oxidation stability 

Peroxides are formed by the oxidation of fatty acids in oils, these can have adverse effect on quality of oils and food products. Proxidant metals, such as copper and iron, and light, temperature and sensitizers can promote oxidation process. Olive oils have high stability to oxidation and are fit to consume with PV values up to 15 meq O_2_/kg oil^21^, but due to its high levels of polyunsaturated fatty acids, linseed oil oxidizes very fast.

In this study, storage period had significant (p<0.05) effect on increasing PV of all samples (Table 1). As expected, oil blends with a higher percent of linseed oil in them showed increased PV during storage (Table 1). However, on the first day, formulated oil containing 5% linseed oil had the highest PV but had a good stability during storage, which could be related to its high olive oil content.

The Rancimat test showed 12.48-, 9.66- and 8.13-hour stabilities for oils with 5, 10 and 15% linseed oil, respectively. As expected, samples with higher content of linseed oil indicated lower oxidative stability, which was in agreement with PVs of all the oil samples. Regarding the oxidative stability of the oil blends, blends with lower linseed oil content (5%) could be used as cooking oil but other blends (with 10 and 15 % of linseed oil) could be also used as salad oils.

### Fatty acid profile 

The fatty acid composition of vegetable oils is responsible for their physical, chemical and nutritional quality. Therefore, it could be considered as one of the most important parameters for oils and fats. The achieved data for fatty acid compositions in linseed, sesame and olive oils were in agreement with the previous literature.^25^ According to the findings, linseed oil with its high levels of linolenic acid (55%) was a rich source of ω_3_ fatty acids, but olive and sesame oils were relatively poor in this fatty acid (Table 2). Blending these oils lead to significant difference (p < 0.05) in fatty acid profile and by increasing linseed oil percent in the mixture, the ω_3_ level increased considerably (Table 2).

**Table 2:** Fatty acid composition (%) and nutritional quality of pure oils and their blends (mean ± SD).

-	**Pure oils**			**Olive: sesame: linseed mixtures**		
	**Linseed oil**	**Sesame oil**	**Olive oil**	**65:30:5**	**60:30:10**	**55:30:15**
C16:0	6.7 ± 0.20	10.7 ± 0.2	13.2 ± 0.15	12.1 ± 0.3	11.6 ± 0.1	11.6 ± 0.2
C18:0	2.5 ± 0.4	6.5 ± 0.4	4.0 ± 0.3	4.0 ± 0.5	4.13 ± 0.15	3.8 ± 0.3
C20:0	0.2 ± 0.05	0.25 ± 0.2	0.6 ± 0.05	0.2 ± 0.05	0.2 ± 0.1	0.2 ± 0.1
C24:0	-	-	0.5 ± 0.02	-	-	-
Total SFA	9.43 ± 0.15	17.4 ± 0.3	18.9 ± 0.4	16.3 ± 1	16.9 ± 0.4	14.6 ± 0.3
C18:1 (ω_9_)	20.3 ± 0.3	41.8 ± 0.2	69.1 ± 0.1	58.8 ± 0.8	54.2 ± 0.2	51.4 ± 0.1
C16:1	-	-	3.0 ± 0.1	2.0 ± 0.05	2.0 ± 0.3	1.5 ± 0.2
Total MUFA	20.0 ± 0.88	41.8 ± 0.1	72.03 ± 0.2	58.8 ± 0.8	54.2 ± 0.2	51.4 ± 0.1
C18:2 (ω_6_)	12.90 ± 0.4	40.09 ± 0.05	11.4 ± 0.2	22.2 ± 0.1	23.9 ± 0.2	35.3 ± 0.3
C18:3 (ω_3_)	57.1 ± 0.2	0.8 ± 0.2	1.2 ± 0.1	4.3 ± 0.1	8.2 ± 0.2	11.4 ± 0.4
Total PUFA	70 ± 0.2	40.9 ± 0.4	12.6 ± 0.3	26.5 ± 0.3	32.1 ± 0.1	35.3 ± 0.3
PUFA:SFA	7.42 ± 0. 2	2.35 ± 0.2	0.66 ± 0.04	1.62 ± 0.02	1.8 ± 0.3	2.41 ± 0.4
ω_6_:ω_3_ ratio	0.22 ± 0.01	50.1 ± 0.1	8.46 ± 1.5	5.1 ± 0.1	2.9 ± 0.2	2.0 ± 0.5
HH^1^	13.24 ± 0.11	7.72 ± 0.02	6.14 ± 0.1	6.88 ± 0.3	7.20 ± 0.2	8.03 ± 0.03
AI^2^	0.00 ± 0.1	0.13 ± 0.3	0.15 ± 0.04	0.14 ± 0.2	0.13 ± 0.03	0.12 ± 0.1
TI^3^	0.04 ± 0.01	0.26 ± 0.35	0.38 ± 0.03	0.30 ± 0.1	0.26 ± 0.1	0.20 ± 0.1

For treatments see Table 1. ^1 ^Hypercholesterolemic ratio , ^2^Atherogenic index , ^3^Thrombogenic index

### Nutritional properties

The nutritional quality of linseed, sesame and olive oil blends were evaluated by nutritional indexes such as AI, TI, HH, PUFA: SFA and the ω_6_:ω_3_ ratio_._ These indexes all evaluate the nutritional quality of foods based on their fatty acid compositions.

AI and TI can be used as predictors or risk factors for cardiovascular diseases. Thus, these indexes should be kept at low levels in a healthy daily diet. AI and TI were lower than one in all three treatments due to their high PUFA content. These results were in agreement with Guimaraes et al. for levels of AI and TI for sesame and linseed oil.^26^

The HH index indicated the fatty acids effects on cholesterol metabolism and high level of this index is important from a nutritional point of view. The HH value in the treatments containing 5, 10 and 15% linseed oil were 6.88, 7.2 and 8.03, respectively‏.

Low level of PUFA: SFA ratio in diets (below 0.45) is a risk factor for increased blood cholesterol levels.^27^ In the present study, PUFA: SFA ratio in linseed oil was very high (7.42) and increasing its content in mixtures lead to significant increase (p<0.05) in this ratio for the formulated samples. PUFA: SFA ratios in 5, 10 and 15% linseed oil were 1.62, 1.8 and 2.41, respectively. Thus, these results indicated that these blended oils have suitable and balanced fatty acid compositions.

Because of essential fatty acid and bioactive compounds importance in health, food products are enriched and fortified with oils and oilseeds with high essential fatty acids and bioactive compounds content.^28,29,12^ Essential fatty acids and their balanced ratio have important health effects, therefore, should be considered in our daily diet. The optimal ratio of ω_6_:ω_3_ fatty acids for the cure or prevention of diseases was defined as 1:1 to 4:1.^5^ This ratio in linseed, sesame and olive oils were 0.22, 9.5 and 50.1, respectively. Therefore, these oils in the pure form do not have optimal ω_6_:ω_3_ ratios.

The addition of linseed oil due to its high levels of linolenic acid lead to an effective improvement in the ω_6_:ω_3_ ratio of the pure oils (Table 2). The levels of the ω_6_:ω_3_ ratio in 65:30:10 and 60:30:15 were balanced and in agreement with the literature (Table 2).^5^

Overall, all the five nutritional indexes for quality evaluation indicated that these formulated oils have good nutritional profiles and could have positive health effects for consumers.

### Rheological properties

**Figure 1:**
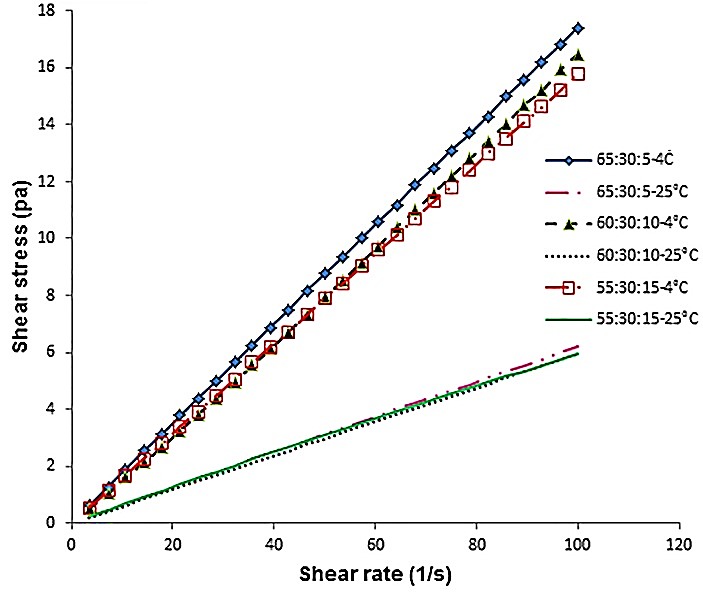
Relation between shear stress and shear rate of blended oils at 4 °C and 25°C.
Treatments are olive, sesame and linseed oils in three ratios of 65:30:5; 60:30:10; and 55:30:15, respectively.

Rheology and viscosity properties are easy to measure in the food industry and their results can be used to determine properties and behavior of suspensions, solutions and mixtures. Rheological properties of oils can be important in food formulation and also during processing.^30^ Figure 1, shows the obtained results from triplicate measurements for shear stress and shear rates of the blended oils at 4°C and 25°C. At both temperatures, all three blended oils showed a linear relationship between the shear stress and shear rate, which meant that these oils have a typical Newtonian behavior. Therefore, the determination viscosity and regression coefficient (R^2^) was carried out using Newton’s law equation (Eq. 4).

**Figure 2:**
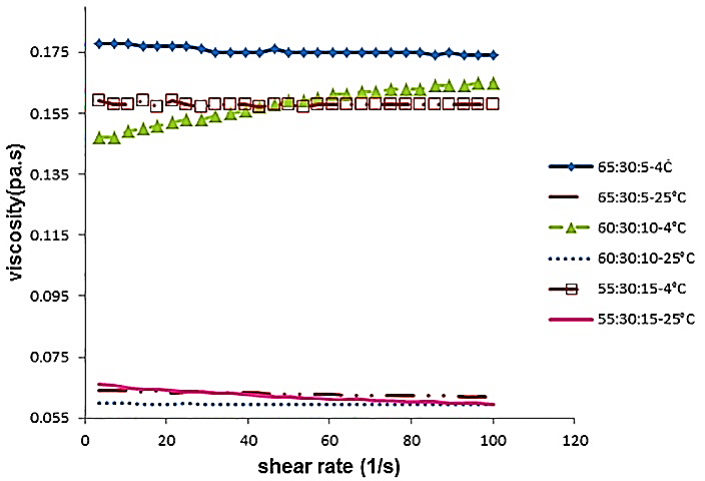
Relation between Viscosity and Shear Rate of blended oils at 4 °C and 25°C.
For treatments see Figure 1.

The viscosity of the formulated oils remained generally constant at all shear rates within the 90% confidence interval (Figure 2). Thus, this confirmed that these oils followed Newtonian behavior at speeds from 0-100 (1/s). Fresh vegetable oils, due to their long chain molecules, also show Newtonian flow behaviors.^12^

As expected, the viscosity of the blended oils at 25°C was significantly lower (p<0.05) than the viscosity measured at 4°C. Increasing the temperature could increase the average speed of the molecules and, because of increasing the frequency of their collisions; the viscosity decreases.^31^ The viscosity at 4°C was about two- to three-fold the viscosity at 25°C; this is important as amount of energy needed for pumping the oils increased considerably and the level of their heat transfer also changed at different temperatures.

Furthermore, it was observed that viscosity had significantly decreased (p<0.05) with increasing linseed oil percent in the mixtures. This can be caused by the high unsaturated fatty acids, specifically, the linolenic acid present in the linseed oil.

Oils with a high level of double bonds in their fatty acid chains show lower viscosity levels because of their weak structures.^32^ Double bonds, due to their space requirements do not allow molecules to be stacked close to each other. Therefore, oils with high amount of unsaturated oils cannot have a rigid and fixed structure and so behave in a more fluid-like way. Thus, there are some negative correlations between the fatty acid composition of some oils and their viscosity.

## Conclusion

Blending linseed oil with sesame and olive oils created a positive nutritional effect with improved stability in formulated oils. Oxidative stability parameters (AV and PV) showed the oil blends have good stability during storage. The Rancimat results showed that the 65:30:5 mixture could be used as a cooking oil, but 60:30:10 and 55:30:15 treatments could be used as salad oils. Furthermore, blended oils had good nutritional indexes, including AI, TI, HH, PUFA: SFA and ω_6_:ω_3_ ratio. All treatments showed Newtonian behavior and increasing temperature, and the content of linseed oil in the mixtures lead to decreases in their viscosity. This study indicated that incorporation of linseed oil with sesame and olive oils can give a functional oil with a balanced ω_6_:ω_3_ ratio, positive levels of bioactive compounds and suitable stability.

## Acknowledgments

Funding for this study was provided by Tabriz University of Medical Sciences and the Department of Food Science and Technology.

## Ethical Issues

Not applicable

## Conflict of Interest

Authors declare no conflict of interest in this study.
